# Beyond Eosinophilia: A Multisystem, Non-eosinophilic Löffler-Like Pulmonary Syndrome From Household Helminthic Transmission

**DOI:** 10.7759/cureus.105989

**Published:** 2026-03-27

**Authors:** Kian Memari, Tara Sanjabi, Sergio A Rodriguez, Joren Manuel, Peter Cohen, Lissette P Lazo

**Affiliations:** 1 Family Medicine, Palmetto General Hospital, Hialeah, USA; 2 Research, Touro College of Osteopathic Medicine Montana, Great Falls, USA; 3 Research, Dr. Kiran C. Patel College of Osteopathic Medicine, Nova Southeastern University, Davie, USA; 4 Family Medicine, Dr. Kiran C. Patel College of Osteopathic Medicine, Nova Southeastern University, Davie, USA

**Keywords:** helminth infection, larva migrans, loeffler’s syndrome, non-eosinophilic pulmonary infiltrates, parasitic lung disease, zoonotic transmission

## Abstract

Löffler’s syndrome is classically described as a transient pulmonary disorder associated with eosinophilic infiltration of the lungs, most commonly triggered by helminthic larval migration. While eosinophilia is considered a defining feature, rare cases lacking peripheral or pulmonary eosinophilia have been reported, complicating recognition and diagnosis. Disseminated helminthic infection with multiorgan involvement is an additional uncommon manifestation that may further obscure clinical identification.

We describe a case of a 40-year-old woman with extensive environmental and animal exposure who developed pulmonary, neurologic, gastrointestinal, and cutaneous symptoms following suspected household zoonotic helminthic transmission. Despite elevated immunoglobulin E (IgE) levels and characteristic clinical features suggestive of larval migration, peripheral eosinophilia was absent. Multiple household members developed similar symptoms, supporting a shared exposure. The patient improved following empiric antihelminthic therapy and coordinated treatment of household contacts and domestic animals.

This case highlights a rare non-eosinophilic variant of a Löffler-like pulmonary syndrome associated with disseminated helminthic infection and underscores the importance of considering parasitic disease in patients with multisystem symptoms and environmental exposure, even in the absence of eosinophilia.

## Introduction

Löffler’s syndrome, also known as simple pulmonary eosinophilia, is a transient pulmonary disorder characterized by respiratory symptoms, migratory pulmonary infiltrates, and peripheral eosinophilia resulting from hypersensitivity reactions to migrating helminth larvae within the lungs [1,2]. The syndrome most commonly occurs during the pulmonary migration phase of parasitic infections such as *Ascaris lumbricoides*, hookworms (*Ancylostoma duodenale* and *Necator americanus*), and *Strongyloides stercoralis* [2,3].

During larval migration through the pulmonary circulation and alveoli, host immune responses trigger eosinophil-mediated inflammation that produces the characteristic pulmonary findings of transient infiltrates and peripheral eosinophilia [1,3]. Eosinophils play a critical role in host defense against helminths through antibody-dependent cellular cytotoxicity and the release of cytotoxic granule proteins [4]. For this reason, eosinophilia is often treated as a key diagnostic clue in suspected parasitic pulmonary disease.

However, eosinophilia is not universally present in helminthic infections. Variability in host immune responses, parasite burden, chronicity of infection, parasite species, and parasite-mediated immune modulation may all contribute to atypical presentations with minimal or absent eosinophilia [4-6]. In such cases, overreliance on eosinophilia may delay recognition of parasitic infection, particularly when the clinical picture is dominated by extraintestinal or extrapulmonary symptoms.

Although Löffler’s syndrome typically manifests as a self-limited pulmonary process, helminthic infections may occasionally produce systemic disease through larval dissemination to other organs. Dermatologic, gastrointestinal, neurologic, and ocular manifestations have been described in zoonotic helminthic infections, especially in the setting of environmental or animal exposure [6,7]. Recognition of atypical, non-eosinophilic presentations is therefore clinically important because diagnosis may depend more heavily on exposure history, symptom clustering, and response to empiric treatment than on a single laboratory marker.

## Case presentation

A 40-year-old woman presented to clinic requesting evaluation after observing visible worms in her stool. One year earlier, she had adopted a stray dog that was subsequently found to have hookworm infection, with delayed veterinary treatment. Shortly thereafter, she developed progressive fatigue, diffuse abdominal pain, increased appetite, pruritic dermatoses, and a persistent cough associated with a foreign body sensation in the upper airway.

She also reported episodic visual disturbances described as aberrant linear gray shapes moving across her visual field, as well as new-onset seizures. Cutaneous findings progressed to include a serpiginous rash on the right ankle and lymphatic tracking of the upper extremities bilaterally. Over time, similar symptoms developed in multiple household members.

**Figure 1:**
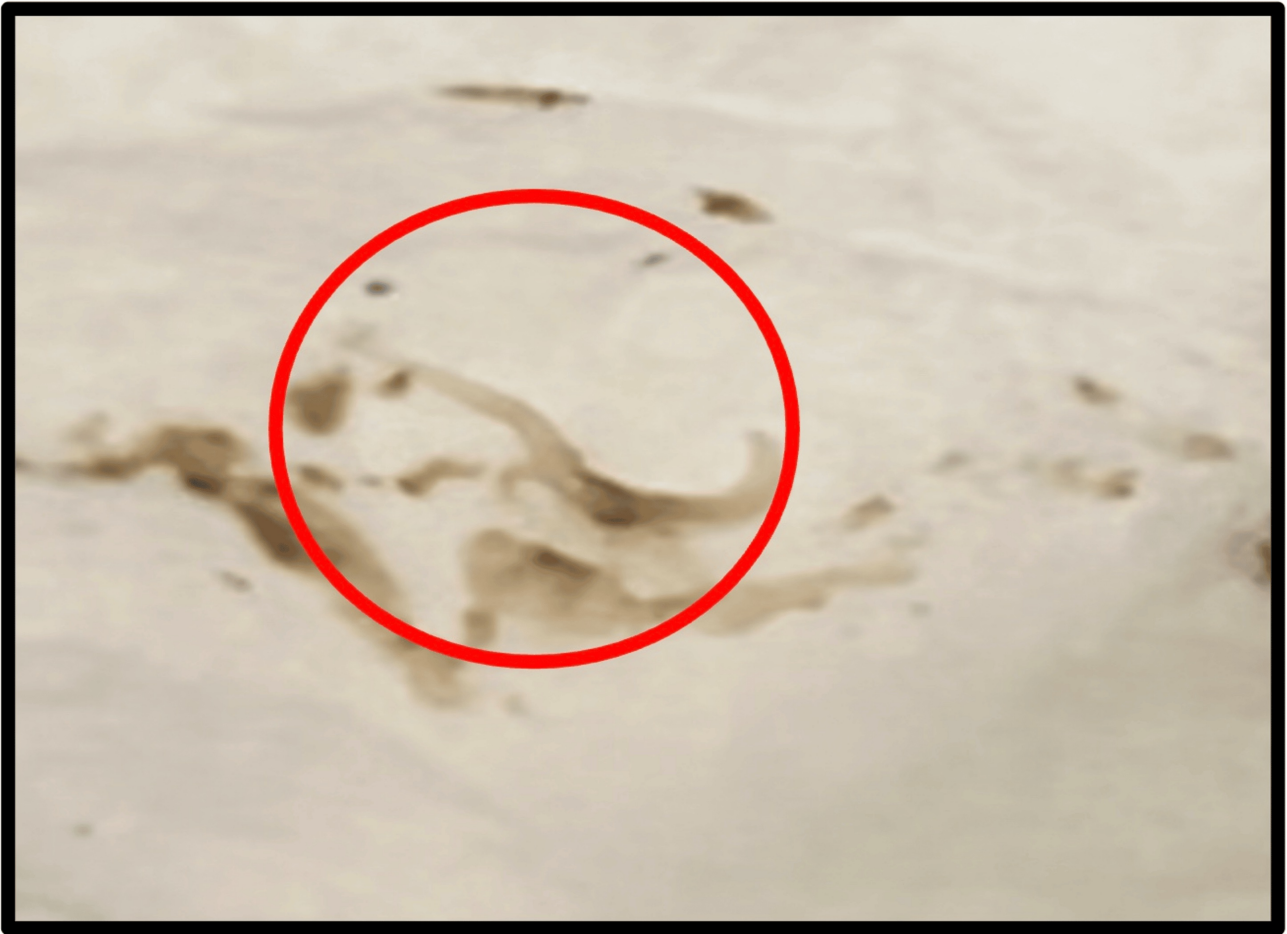
Visual inspection of helminth manually extracted from stool; gross examination demonstrated approximately 1 cm curved worms with sharply hooked ends

Her social history was notable for employment as a zookeeper and frequent adoption of stray dogs and cats. A home-based tape test was negative for ova or parasites. In clinic, several helminths were manually extracted from stool; gross examination demonstrated approximately 1 cm curved worms with sharply hooked ends, morphologically suggestive of hookworm species (Figure 1). Formal microscopic speciation and parasitology consultation were not performed, so definitive species confirmation was not established.

Initial laboratory evaluation revealed elevated total immunoglobulin E (IgE) at 169 kU/L, mild transaminitis, hyponatremia, and hyperlipidemia. Peripheral eosinophil counts remained within normal limits (WNL). Stool ova and parasite testing, including Giardia antigen, was negative. Serologic testing for Strongyloides and Toxocara immunoglobulin G antibodies was also negative (Table 1). Repeat stool concentration techniques and serial stool sampling were not performed.

**Table 1:** Summary of laboratory results at initial evaluation Calc: Calculated; MCH: Mean corpuscular hemoglobin; MCHC: Mean corpuscular hemoglobin concentration; MCV: Mean corpuscular volume; MPV: Mean platelet volume; RDW: Red cell distribution width; eGFR: Estimated glomerular filtration rate; BUN: Blood urea nitrogen; WNL: Within normal limits; IgE: Immunoglobulin E; ALT: Alanine aminotransferase; AST: Aspartate aminotransferase

Parameter	Patient's value	Unit	Normal reference range	Clinical note/interpretation
Hemoglobin A1c	5.9	% of total hemoglobin	<5.7	High
IgE	169	kU/L	≤114	High
Absolute basophils	50	cells/uL	0-200	WNL
Absolute eosinophils	82	cells/uL	15-500	WNL
Absolute lymphocytes	1241	cells/uL	850-3,900	WNL
Absolute monocytes	460	cells/uL	200-950	WNL
Absolute neutrophhils	4467	cells/uL	1,500-7,800	WNL
Basophils	0.8	%	-	-
Eosinophils	1.3	%	-	-
Hematocrit	48.5	%	38.5-50.0	WNL
Hemoglobin	16	g/dL	13.2-17.1	WNL
Lymphocytes	19.7	%	-	-
MCH	29.2	pg	27.0-33.0	WNL
MCHC	33	g/dL	32.0-36.0	WNL
MCV	88.5	fL	80.0-100.0	WNL
Monocytes	7.3	%	-	-
MPV	9.5	fL	7.5-12.5	WNL
Neutrophils	70.9	%	-	-
Platelet count	277	10^3^/uL	140-400	WNL
RDW	13.1	%	11.0-15.0	WNL
Red blood cell count	5.48	10^6^/uL	4.20-5.80	WNL
White blood cell count	6.3	10^3^/uL	3.8-10.8	WNL
Alblumin	4.7	g/dL	3.6-5.1	WNL
Alblumin/globulin ratio	1.7	(calc)	1.0-2.5	WNL
Alkaline phosphate	87	U/L	36-130	WNL
ALT	48	U/L	9-46	Elevated
AST	21	U/L	10-40	WNL
Bilirubin, total	1.5	mg/dL	0.2-1.2	Elevated
Calcium	9.5	mg/dL	8.6-10.3	WNL
Carbon dioxide	21	mmol/L	20-32	WNL
Chloride	99	mmol/L	98-110	WNL
Creatinine	0.87	mg/dL	0.60-1.29	WNL
eGFR	108	mL/min/1.73 m^2^	≥60	WNL
Globulin	2.8	g/dL (calc)	1.9-3.7	WNL
Glocuose	93	mg/dL	65-99	Fasting reference interval
Potassium	4.2	mmol/L	3.5-5.3	WNL
Protein, total	7.5	g/dL	6.1-8.1	WNL
Sodium	130	mmol/L	135-146	Low
BUN	9	mg/dL	7-25	WNL

Given the visual symptoms, the patient was referred for ophthalmologic evaluation for possible ocular larva migrans. Given the reported seizures, systemic larval migration involving the central nervous system remained a diagnostic consideration; however, neurologic imaging was not performed prior to empiric treatment. These factors were considered in the multidisciplinary evaluation, but definitive ocular or neurologic confirmation was not obtained.

Because of the high clinical suspicion for disseminated helminthic infection, empiric albendazole therapy was initiated, and the patient was referred to infectious diseases and gastroenterology. Following treatment, she reported marked improvement in systemic symptoms and described coughing up intact worms for several days after beginning therapy.

One household member initially declined treatment and later re-presented with recurrent symptoms, supporting ongoing household exposure and possible reinfection. After coordinated retreatment with albendazole and simultaneous treatment of household contacts and domestic animals, sustained symptom resolution was achieved.

## Discussion

Löffler’s syndrome represents a pulmonary hypersensitivity reaction to helminthic larval migration and is classically characterized by respiratory symptoms, transient pulmonary infiltrates, and peripheral eosinophilia [1,2]. This case is unusual because the pulmonary component of the illness occurred in the absence of peripheral eosinophilia, yet was accompanied by elevated IgE, serpiginous cutaneous lesions, gastrointestinal symptoms, visual complaints, neurologic symptoms, household clustering, and symptomatic improvement after antihelminthic therapy. Taken together, these features supported a clinically suspected helminthic larval migration syndrome despite nondiagnostic standard testing.

The absence of eosinophilia does not exclude helminthic infection. Eosinophil responses vary according to parasite burden, phase of infection, host immune response, and organism-specific immune modulation [4-6]. In some patients, eosinophilia may be minimal, transient, or absent, particularly when infection is chronic, localized, or immunologically atypical [4,5]. Elevated IgE in this patient provided an additional clue supporting type 2 immune activation despite normal eosinophil counts, reinforcing the concept that eosinophilia should not be treated as an obligatory diagnostic requirement in suspected helminthic disease [4].

This case also highlights the important diagnostic limitations of stool and serologic testing. Single stool ova and parasite examinations may have limited sensitivity because egg shedding can be intermittent and parasite burden may be low, particularly early in infection or during migratory phases before mature intestinal egg production [3,6,8,9]. Concentration techniques and repeated sampling can improve detection, but even these approaches may remain imperfect [8,9]. Similarly, serologic assays may vary in sensitivity according to parasite species, tissue tropism, and stage of infection, and a negative result does not reliably exclude disease when clinical suspicion is high [6,9]. In the present case, only a single formal stool examination was documented, and repeat concentration-based studies were not performed, which likely limited diagnostic yield.

The visual disturbances and seizures raised concern for possible systemic larval migration, including ocular or neurologic involvement. Zoonotic helminthic infections such as toxocariasis can involve the eye and central nervous system and may present with variable or nonspecific findings [6,7,10]. Because ophthalmologic findings and neuroimaging were not ultimately available in this case, these manifestations remained clinically suspected rather than objectively confirmed. This is an important limitation, but it does not negate the broader diagnostic pattern, especially given the multisystem presentation and treatment response.

Definitive species identification was also not established. The extracted specimen underwent gross visual inspection only, and no formal microscopic parasitology consultation was obtained. Although the curved morphology with a hooked anterior end was suggestive of hookworm species, the diagnosis remained syndromic rather than species-confirmed. This limitation is relevant because different zoonotic helminths can produce overlapping pulmonary, cutaneous, gastrointestinal, ocular, and neurologic manifestations [3,6,7].

**Figure 2:**
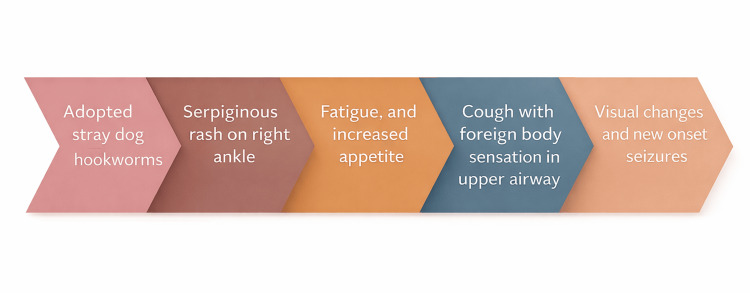
Chronologic progression of key symptoms in patient’s presentation with helminthic infection Chronologic depiction of symptom development in the index patient and household context, illustrating pulmonary, gastrointestinal, dermatologic, neurologic, and visual manifestations followed by clinical improvement after coordinated antihelminthic therapy. This figure was created by the authors using Microsoft PowerPoint (Microsoft Corporation, USA). No external copyrighted materials or artificial intelligence tools were used.

From a management perspective, this case underscores two practical points. First, empiric antihelminthic therapy may be reasonable when exposure history, symptom pattern, and supportive findings strongly suggest helminthic infection despite negative initial testing, particularly when repeated or specialized diagnostic testing is not readily available (Table 2). Second, evaluation and coordinated treatment of household contacts and animal reservoirs are critical when symptoms cluster within a shared environment, because untreated contacts or pets may perpetuate exposure and reinfection. The recurrence of symptoms in an untreated household member in this case strongly supported this principle. Figure 2 provides a practical framework linking exposure history, atypical laboratory findings, multisystem features, empiric therapy, and household management.

**Table 2:** Common clinical manifestations of helminthic disease by organ system

Anatomic Location	Clinical Manifestations
Lungs	Loeffler’s syndrome, dry cough, wheezing, dyspnea, and transient pulmonary infiltrates
Gastrointestinal tract	Abdominal pain, nausea, steatorrhea, intestinal obstruction, occult blood loss/anemia, and rectal prolapse
Liver and biliary tree	Hepatomegaly, right upper quadrant pain, biliary obstruction, cholangitis, and liver abscesses
Skin	Cutaneous larva migrans, pruritus, urticaria, and “swimmer’s itch”
Central nervous system	Neurocysticercosis, meningitis, and focal neurological deficits
Heart	Myocarditis and pericarditis
Muscles	Myalgia, muscle edema, and weakness
Lymphatic system	Lymphedema, hydrocele, and elephantiasis
Bladde/urogenital	Hematuria, dysuria, and increased risk of bladder squamous cell carcinoma

## Conclusions

This case describes a rare non-eosinophilic, multisystem Löffler-like pulmonary syndrome associated with suspected household zoonotic helminthic transmission. It highlights the limitations of relying on eosinophilia and single-test diagnostic strategies alone in parasitic disease.

Clinicians should maintain a high index of suspicion for helminthic infection in patients with compatible environmental or animal exposure and multisystem symptoms, even in the absence of eosinophilia. Early empiric antihelminthic therapy, repeat or specialized diagnostic evaluation when available, and coordinated treatment of household contacts and animal reservoirs may be important for symptom resolution and prevention of reinfection.
